# Evaluation of Surface Finishing Efficiency of Titanium Alloy Grade 5 (Ti–6Al–4V) After Superfinishing Using Abrasive Films

**DOI:** 10.3390/ma17215198

**Published:** 2024-10-25

**Authors:** Katarzyna Tandecka, Wojciech Kacalak, Michał Wieczorowski, Thomas G. Mathia

**Affiliations:** 1 Department of Engineering and Informatics Systems, Faculty of Mechanical Engineering and Energy, Koszalin University of Technology, 75-620 Koszalin, Poland; wojciech.kacalak@tu.koszalin.pl; 2 Faculty of Mechanical Engineering, Institute of Applied Mechanics, Poznan University of Technology, 3 Piotrowo St., 60-965 Poznan, Poland; michal.wieczorowski@put.poznan.pl; 3 Laboratoire de Tribologie et Dynamique des Systemes (LTDS), Ecole Centrale de Lyon, Centre National de la Recherche Scientifique, 69134 Lyon, France

**Keywords:** surface finishing, abrasive films, finishing, Grade 5, superfinishing, titanium alloy, surface finishing efficiency

## Abstract

Ti–6Al–4V is the most commonly used alpha–beta titanium alloy, making it the most prevalent among all titanium alloys. The processed material is widely employed in aerospace, medical, and other industries requiring moderate strength, a good strength-to-weight ratio, and favorable corrosion resistance. A microfinishing process on the titanium alloy surface was conducted using abrasive films with grain sizes of 30, 12, and 9 μm. Superfinishing with abrasive films is a sequential process, where finishing operations are performed with tools of progressively smaller grains. The surface topography measurements of the workpiece were taken after each operation. The experiment was in the direction of developing a new surface smoothness coefficient considering the number and distribution of contact points so as to properly evaluate the quality of the surface finishing. The results showed that the finest-grain films gave the most uniform contact points, thus offering the best tribological characteristics; the 9 LF (micron lapping film) tools gave the smoothest surfaces (Sz = 2 µm), while the biggest-grain films, such as the 30 FF (micron microfinishing film), were less effective since large protrusions formed. This is a suitable study to explore the optimization paths for the superfinishing of titanium alloys, with implications for improving the performance and longevity of components in critical industrial applications.

## 1. Introduction

Among all titanium alloys, titanium alloy Grade 5, popularly known as Ti–6Al–4V, is by far the most utilized. This is so because it possesses an unbeatable combination of properties: its strength-to-weight ratio and resistance to corrosion and high temperatures. Such a unique combination places the material in high demand within industries that operate under critical conditions, such as the aerospace, biomedical, and automotive industries. Due to its poor surface finish, the use of Ti–6Al–4V, particularly in applications requiring smooth surfaces for meeting critical performance and durability requirements, is difficult to achieve, despite its excellent mechanical properties. In light of this, surface finishing, a superfinishing process, is highly vital in view of the enhancement of the tribological properties of components made from titanium alloys. 

The abrasive machining of titanium alloys, for example those of Ti–6Al–4V, is a stochastic process [1,2,3]. It is also a random process in nature, where individual grains are geometrically different from each other and may reach the surface of the material in any random fashion. The stochastic nature of abrasive machining is what further complicates managing parameters such as the homogeneity of the material removal, the micro-protrusions formed, and hence the quality of the surface finish. Abrasive machining is dynamic, with variables including pressure, cutting speed, and the nature of the abrasive grains. All these variables affect the material removal efficiency and final surface quality. In the case of Ti–6Al–4V, all these processes are further complicated by properties such as those of this material [4,5,6]. Because of its high tensile strength and low thermal conductivity, high temperatures develop in the cutting zone during abrasive machining. In this respect, low thermal conductivity means that the material is prone to hot spot formation and is characterized by a high tendency for plastic deformation and the adhesion of the workpiece material on the tool surface, in addition to micro-cracking [7,8,9,10,11]. These phenomena negatively affect the quality of the surface finish and may, moreover, accelerate wear.

One of the major problems with the abrasive machining of Ti–6Al–4V alloy is its tendency to adhere on the surface of the abrasive tool. During this process, the particles of the workpiece material may stick to the grains of the abrasive tool and hence clog up the tool, thereby reducing its efficiency. This phenomenon makes it not only more difficult to achieve the desired surface quality, but it also increases friction, generating extra heat and further deteriorating the thermal problems in machining titanium. Another significant problem deals with chip formation [12]. Chips of titanium are usually thin and ribbon-like, which can break and change their influence on the machined surface in quite unpredictable ways [10,13]. If not managed properly, these chips can scratch the surface deeply, which calls for additional finishing steps [12,13,14]. The coolant condition has also demonstrated a high level of sensitivity in the case of the abrasive machining process of Ti–6Al–4V [15]. Insufficient coolants or the improper selection of cooling fluid may heat up the material and bring about micro-cracks and degradation in the mechanical properties of the surface [16,17,18]. Thus, cooling and lubricating fluids are a must in the case of machining titanium alloys, as these reduce not only the temperature but also the friction and help to clean the debris on the tool surface [19]. The abrasive machining of Ti–6Al–4V, though a stochastic process, can be quite effectively optimized by the proper selection of tools, process parameters, and cooling strategies. This is because of the unique properties of titanium, such as its low thermal conductivity and an affinity for adhesion. By using the right method, it is achievable to obtain a high-quality surface finish, which is crucial for the demands of industrial applications.

Among the many grades of titanium alloys, Grade 5, more correctly referred to as Ti–6Al–4V, is by far the most popular for its unparalleled combination of properties. It is an alloy of titanium, containing 6% aluminum, 4% vanadium, iron to a maximum of 0.40%, oxygen to a maximum of 0.20%, and carbon to a maximum of 0.08%. This specific composition endows the alloy with a very excellent strength-to-weight ratio and good resistance to corrosion, and it hence finds wide uses in industries such as aerospace, automotive, medical, and offshore oil and gas industries. Its mechanical properties include yielding at 931 MPa, while the tensile strength for small diameters is 862 MPa, with the elongation at 10%. Ti–6Al–4V can be used at temperatures of up to 400 °C, with a melting point of 1648 °C. It is highly formable and readily weldable, and thus lends itself to a wide variety of applications.

**Figure 1:**
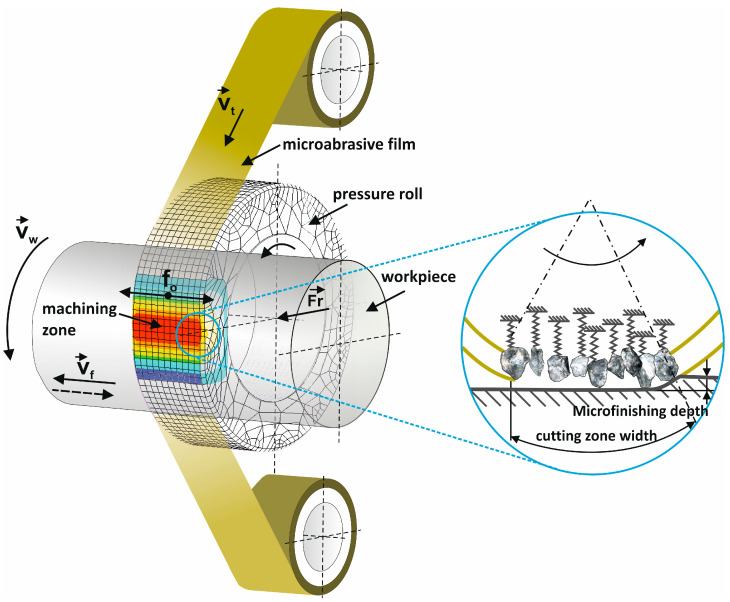
Kinematic schematic of rotary surface finishing with lapping films, showing the specified quantities on the diagram: *v_t_*—tool speed; *v_w_*—workpiece speed; *v_f_*—tool feed speed; *f_o_*—tool oscillation frequency; and *F_r_*—the pressure force of the pressing roller [42].

The above features of the machining of Ti–6Al–4V, characterized by the adhesion to the tool, heating in the zone of cut, and low quality of the surface, can be considerably improved by applying a sequential superfinishing process using abrasive films. Superfinishing is a process of advanced finishing that enables attaining surfaces with very low roughness [2,20,21], which is essential in many critical applications related to aerospace and medical fields. Another important feature of this process is that the tool, an abrasive film, is used only once on the given workpiece to ensure a uniform abrasive efficiency and to minimize tool wear [22,23]. Apart from that, the tool travels much slower compared to the workpiece, which provides better control over the surface finishing process [24,25]. Hence, the methodology of pressing an abrasive film against the workpiece surface is a crucial aspect of the process [26,27,28]. With superfinishing, unlike in any conventional machining process, the abrasive film is pressed by an elastic element to ensure that the pressure is equally distributed over the whole area of contact (Figure 1) [29,30,31,32]. 

**Figure 2:**
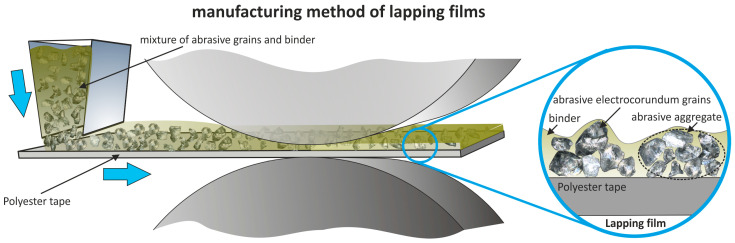
The production scheme of lapping films [42].

This reduces the possibility of a form of localized surface damage and permits the cutting process to be under more controlled, gentle conditions [33,34,35]. This elasticity is all the more important because the working of titanium alloys is rather problematic in view of their high strength and low thermal conductivity [36,37,38,39]. The abrasive films used in the process are designed to meet the demands of superfinishing. They are made by applying a layer of binder and abrasive grains onto a polyester backing, embedding the abrasive grains in adhesive, which forms characteristic abrasive aggregates (Figure 2) [40,41]. This construction ensures the uniform distribution of the grains on the tool surface, with the effect of an even material removal from the workpiece. A very accurate surface finish, which is required in those parts that go into applications requiring low roughness and high resistance to wear, results. The following sequential superfinishing application, using the abrasive film, resulted in smooth surfaces aside from overcoming the issues of heat generation and adhesion. This process, due to its precision and controlled cutting, remains ideal for the machining of difficult materials like Ti–6Al–4V and helps in extending components’ lives by improving their operational properties.

Superfinishing is generally a multistep process wherein the refinement of a surface progressively takes place with abrasive films of decreasing grain size [43,44,45]. This not only reduces surface roughness but also enhances surface integrity, which is very much required under high-precision and wear-resistant conditions [46,47]. In the present investigation, four varieties of abrasive films, including 30 FF, 30 LF, 12 LF, and 9 LF, were used to understand their effectiveness with a view to minimize surface roughness and enhance surface quality subsequent to the superfinishing of Ti–6Al–4V. The above-mentioned films were screened for their effectiveness through different advanced techniques like SEM and surface topography analysis. In this way, the investigation here proposes a new coefficient of surface smoothness associated with the number and distribution of the contacting surface points, giving a more realistic interpretation of the finishing process. The innovation of this manuscript is focused on the development of a new coefficient that is specifically designed to quantify smoothness. This coefficient provides a more accurate and applicable representation of the number and distribution of contact points residing on the finished surface. This approach goes beyond the conventional amplitude-based methods that have been commonly used for surface characterization, and it further represents a more complete and practically useful metric in order to improve the process. The approach helps not only in enhancing the control of the processes, but also allows the easy identification of specific tools or operations that could be excluded with little sacrifice in the finish integrity. This research work was undertaken to identify the most appropriate conditions for superfinishing titanium alloy Grade 5 by improving the surface characteristics relevant to its applications in high-performance purposes. This study contributes not only to improved surface finishing processes but also to the greater cause of machining technologies for titanium alloys too, where the surface quality provides a clue to the functionality and durability in service. Due to these challenges, the present study focuses on developing a methodology for evaluating surface smoothness, which, in combination with standardized parameters [48], will provide a broader context for understanding future operational properties. This research aims to improve the understanding of superfinishing processes in the context of titanium alloys, and the results obtained may be crucial for industries where the performance and reliability of components are critical.

## 2. Materials and Methods

### 2.1. Assessment of the Surface Texture of Abrasive Films

The topography of the abrasive films’ surfaces was made using an Olympus LEXT OLS4000 confocal microscope, Tokyo, Japan. The nominal grain sizes of the different films studied were 30 μm (30 FF microfinishing film), 30 μm (30 LF lapping film), 12 μm (12 LF lapping film), and 9 μm (9 LF lapping film). The area of measurement was 256 × 256 µm, with surfaces made up of 1024 by 1024 points. A 50× magnification objective lens was used for the measurements. The abrasive tools used in the study are from a commercial manufacturer, 3 M (St. Paul, MN, USA).

### 2.2. Process of Machining with Abrasive Films

**Figure 3:**
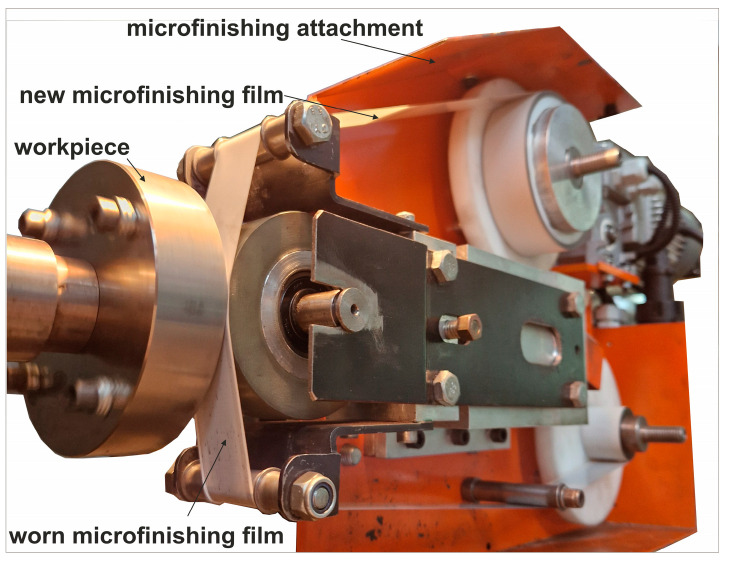
Research setup for the superfinishing process using abrasive films.

The superfinishing process was carried out for a total of 360 s. Machining with abrasive films is characterized as a sequential process. The surface, after conventional grinding, was prepared for the superfinishing process using an abrasive film where the abrasive grains were deposited in an electromagnetic field. The grains were not bonded with a binder but were sharper and vertically oriented due to the production method. The abrasive film used is referred to as a microfinishing film, designated as 30 FF, as the nominal grain size is 30 μm. The process was then continued using an abrasive film called “lapping film” by the manufacturer, where the abrasive grains form aggregates and are embedded in a binder (Figure 2). Every stage took 60 s, which means that the general superfinishing duration was 240 s. The superfinishing treatment was performed by using the GW1 microfinishing attachment (Figure 3). The work material used was titanium alloy Grade 5 (Ti–6Al–4V). The applied roller presser had a hardness of 50 Shore degrees with a pressing force of 50 N. The abrasive film speed was 160 mm/min; the rotational speed of the workpiece was 10 m/min. Also, the tool oscillated at a frequency of 80 Hz. The aforementioned standard machining parameters for the superfinishing are listed in Table 1.

The chemical composition of the machined surfaces was analyzed using EDS on the Phenom ProX tabletop electron microscope, Phenom-World BV, Eindhoven, The Netherlands.

### 2.3. Evaluation of Surface Smoothness After the Superfinishing Process

**Figure 4:**
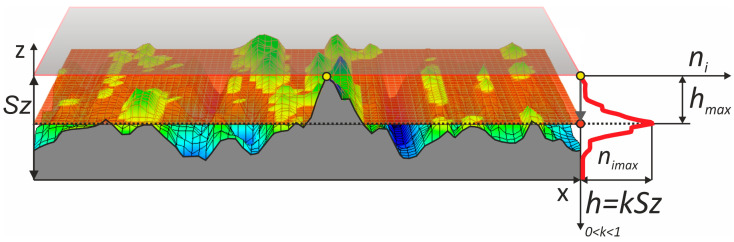
Approach for surface assessment through the “island analysis” method [50].

The measurements of the machined surface were performed in a 3D setup using the Olympus LEXT OLS4000 confocal microscope. After each subsequent machining process with a new tool, the surface was measured in three randomly selected locations. Digital Surf TalyMap Platinum 7.4 software was used for the 3D system analysis of the machined surfaces, and a new method of surface smoothness evaluation, namely, the so-called “island analysis” technique, was proposed in this research work. An island analysis was carried out for each surface, with the cut-off plane being initially placed at the maximum peak and being progressively lowered by a quantity *h* [49], where *h = kSz*, with *Sz* being the maximum height of the surface, until *Sz* was attained. For every cut-off level, *ni* was computed. For each surface, the *h_max_* obtained corresponded to the value for which the *ni* reached a maximum value (Figure 4).

The cut-off plane *h* of the cut-off plane position was investigated within the range 0.0787–2.2813 μm. All the measured surfaces were analyzed. In each analyzed surface, the cut-off plane position was investigated for all the values within the range. Apart from counting the number of islands at each cut-off level *ni*, the perimeter of the base of each elevation at the given level *Pi* was measured, and the arithmetic mean of all the perimeters was calculated. Likewise, the volume of all the islands raised above the cut-off plane *Vi* was computed, and the arithmetic mean of all volumes calculated. Further analysis of the islands entailed determining the highest peak of each island *h_max_*, followed by calculating the arithmetic mean of all the islands elevated above the next cut-off plane. All the determined parameters describing the shape of the islands raised above a specific level were presented in graphs. Since the surface was measured three times after each machining operation, all the obtained results are shown in the form of a graph, where the whiskers indicate the minimum and maximum values, while the curve represents the arithmetic mean of the three measurements. In the article [50], the authors proposed an evaluation of surface smoothness by comparing four variations of machining with the same tool. They introduced a surface smoothness evaluation coefficient based on the number of islands on the smoothed surface and the position of the cut-off plane at the level *h_max_*, where the maximum number of islands *ni_max_* occurs. However, this coefficient needed to be modified, as the previous study focused on the reverse process, where the reference surface was perfectly smooth, and it was deteriorated by the films. In contrast, in the conventional process, where each subsequent tool with a smaller grain size is intended to improve the smoothness of the surface, the surface smoothness evaluation coefficient *c_e_* is expressed by Formula (1).

(1) $$c_{e}=\sqrt{\frac{{ni}_{max}}{\frac{hmax}{Sz}}}$$

### 2.4. Analysis of the Surface of the Worn Abrasive Tool

A Phenom ProX tabletop electron microscope was utilized to investigate the surfaces of the finishing film 30 LF after the superfinishing process, along with the worn tool surfaces and abrasive grains. This SEM (manufactured by Phenom-World BV, Eindhoven, The Netherlands) allowed the detailed observation of the machining remnants on the abrasive film surface. Surface assessments of the worn tool were made directly on the abrasive film.

## 3. Results and Discussion

### 3.1. Analysis of the Unused Abrasive Film Surface

**Figure 5:**
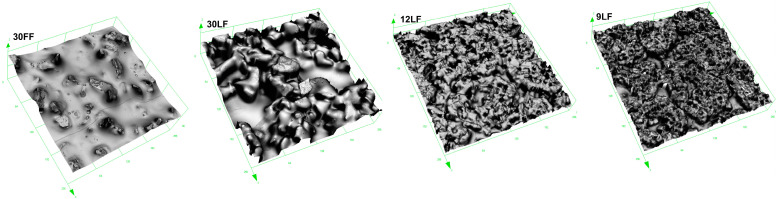
Surfaces of abrasive films with an area of 256 × 256 μm.

This work mapped and documented the topography of the microabrasive tools tested in this research (Figure 5). These tests show a sharp contrast between microfinishing films and superfinishing films since they are far from being similar in structure or function. In the case of microfinishing films, due to their specialized production process, the abrasive grains are positioned directly on the surface of the binder within an electrostatic field. A pre-treatment stage was, therefore, carried out using the selected microfinishing film. This production method promotes the sharpness of the tools, with the abrasive grains optimally oriented in respect to the workpiece. Such an orientation is believed to improve the cutting efficiency and enhance material removal during machining because of the fact that the sharp edges of the grains are maximized in order to interact with the surface. Also, microfinishing films ensure the uniform distribution of grains on the surface for consistent cutting actions over longer periods of time.

The superfinishing films tested in this study have quite different characteristics because of their special manufacturing process. More than 90% of the abrasive grains are enclosed by the binder matrix in these films, and only a small part of the grain is exposed. Due to the deep embedding of the grains, the surface interactions become softer, smoother, and less aggressive; hence, such films are ideal for final finishing stages where only negligible material removal is required. Another distinguishing feature of superfinishing films is that the abrasive grains tend to form characteristic abrasive aggregates. This directly affects the cutting behavior of the film, as the tendency for agglomeration can result in heterogeneity in the material removal. Further, the reduced spacing among the grains in these films limits the space available for the removal of debris and other machining by-products from the processing zone. This problem becomes increasingly critical with the decrease in the nominal size of the abrasive grains. Smaller grains naturally create smaller spacing between the particles and further limit the space for collecting and carrying away debris from the treated surface. The reduced space can facilitate clogging or improper cutting action, especially when large amounts of material have to be removed or extended use is intended. Additionally, the embedding and spacing of grains in a microfinishing film differ from those in a superfinishing film, to which its performance is directly related. Each type of film, therefore, has applications in different stages depending on the results desired in the surface finishing.

### 3.2. Evaluation of Surface Smoothness in the Superfinishing Process Utilizing Abrasive Films

**Figure 6:**
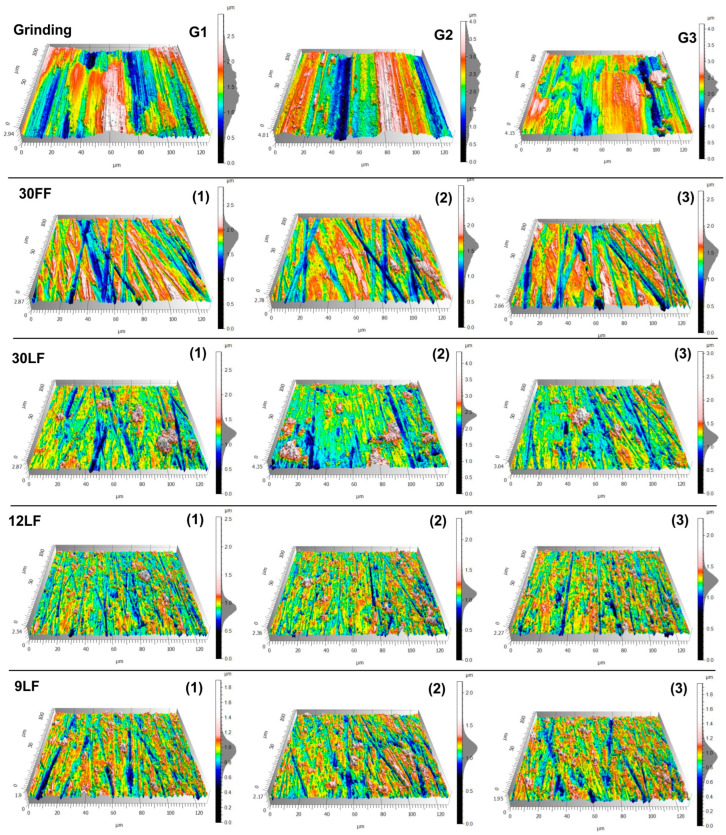
Topographies of the examined titanium alloy surfaces after successive machining operations with abrasive films (30 FF, 30 LF, 12 LF, 9 LF).

After each 60 s of machining, surface measurements were taken in three different locations of the superfinished titanium alloy workpiece. Figure 6 shows the surface topographies of the titanium alloy workpieces after the different stages of the machining, from the grinding process up to the microfinishing and superfinishing operations. The top row (G1–G3) represents the surface after the grinding. It can be seen that surface irregularities and deep scratches are present in a significant way. The microfinishing film of the nominal grain size 30 µm (30 FF) was used in the second row. The texture of the surface shows that the surface roughness decreased by a large amount, with clearer directional patterns. The measurements for locations 1–3 were taken on the machined surface. 

The successive lines represent the surfaces treated by the superfinishing films of the nominal grain sizes 30 µm (30 LF), 12 µm (12 LF), and 9 µm (9 LF), respectively. While the grain size decreases, the surface becomes progressively smoother, which is clearly reflected from the reduced height of the peaks and valleys. The three repeated measurements from each tool that were taken on the surface roughness are shown in columns (1–3) for each type of film. The progression from the grinding to the finer finishing stages demonstrates a significant improvement in the surface uniformity and reduction in the surface irregularities, especially in the final stages using the 9 LF film. The appearance of the surfaces differs at different machining stages. From the grinding after G1 to G3, the surface presents quite aggressive scratches, along with rough peaks. This corresponds to the meaning of high surface irregularity and unevenness, showing deep grooves and a rough texture, which is the result of the coarse machining techniques. The application of the microfinishing film, 30 FF, implements a reduction in the surface irregularities, and the scratches become more linear and uniform. The machining marks are not that deep, and the material tends to distribute more uniformly across the surface. The refinement in the surface topography at this stage is considerably different from the ground surfaces. This process continually smooths and homogenizes the surface as it proceeds with the superfinishing films (30 LF, 12 LF, and 9 LF). The topography of the surface at the 12 LF and 9 LF stages is characterized by much finer and shallower scratches, while the transitions between the peaks and valleys are smoother. These films have finer grains that remove minimal amounts of material, thus leaving a polished and more homogeneous surface. It is clear that as grain size decreases, a reduction in the surface roughness and a more polished texture are exhibited, with an almost flawless surface in the final stages of the superfinishing.

**Figure 7:**
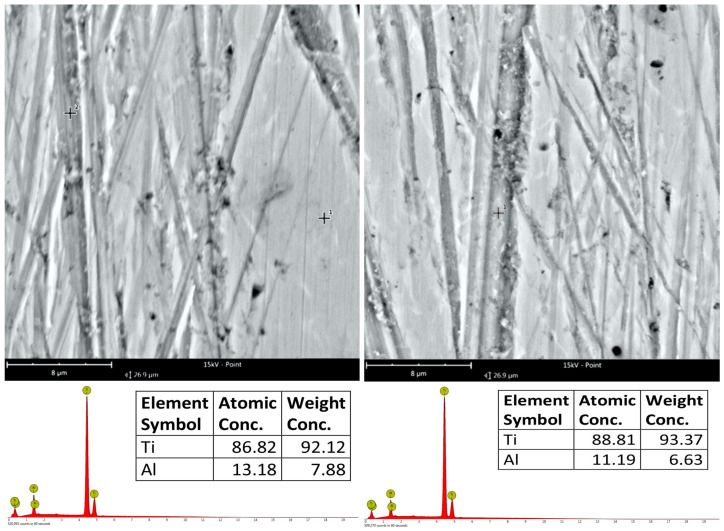
SEM images of the surface processed with 9 LF film, showing clearly intersecting machining marks, and EDS analysis of the composition of these surfaces.

The SEM images of the surface treated by the 9 LF film show crossing machining marks that well distinguish the impact of the abrasive film on the topography of the titanium alloy workpiece (Figure 7). 

The crossing lines indicate a complex interaction between the tool and material with distinct traces from the fine finishing process. The chemical composition of the surface was determined by EDS analysis. The results show the prevalence of titanium, with atomic concentrations in two different locations of 86.82% and 88.81%, while for the aluminum, the atomic concentration is 13.18% and 11.19%, respectively. The weight percentages confirm the high presence of titanium even more, joining together the expected composition of the alloy. These cross-like machining marks constitute some indication, in addition to the EDS results, that the surface had maintained most of its primary elemental structure upon the finishing, while the machining marks suggest the effective yet delicate material removal.

For each measured area, the following surface roughness parameters were computed. The ISO 25178 standard was followed for the computation of these parameters, especially for the height measurements [51]:

- Sa: arithmetical mean height of the surface;
- Sz: maximum height of the surface;
- Sp: maximum peak height;
- Sv: maximum pit height.

**Figure 8:**
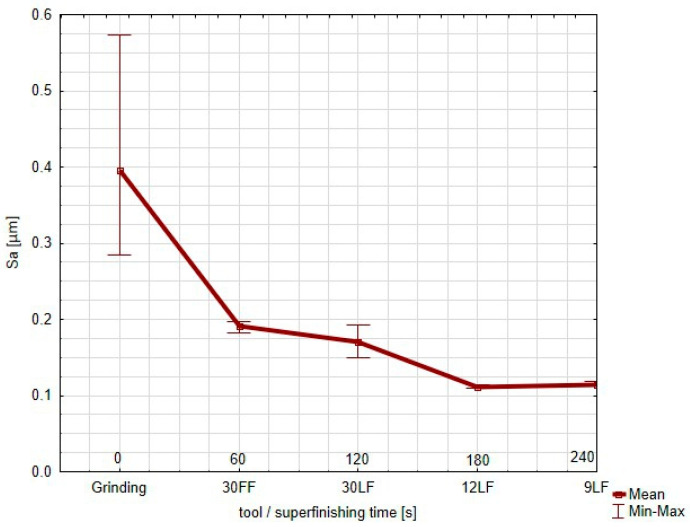
Graph of the *Sa* parameter of the surface after successive smoothing stages.

The graph plots the value of the Sa parameter (Figure 8), namely, the arithmetical mean height of the surface, for every step of the finishing process, from the grinding to the application of increasingly finer superfinishing films (30 FF, 30 LF, 12 LF, and 9 LF). The Sa parameter is an averaging parameter, and its trend is shown as an average; the min–max error bars show the dispersion of the results in the three different measurement locations on the surface. Starting from a high value right after the grinding, which expresses a great surface roughness, the wide min–max bars reflect the great irregularities on the surface. As the process proceeds further with the 30 FF film, the Sa parameter falls sharply, showing quite a drastic reduction in the surface roughness. The min–max bars shrink considerably, indicative of a more consistent finish across the measured locations.

**Figure 9:**
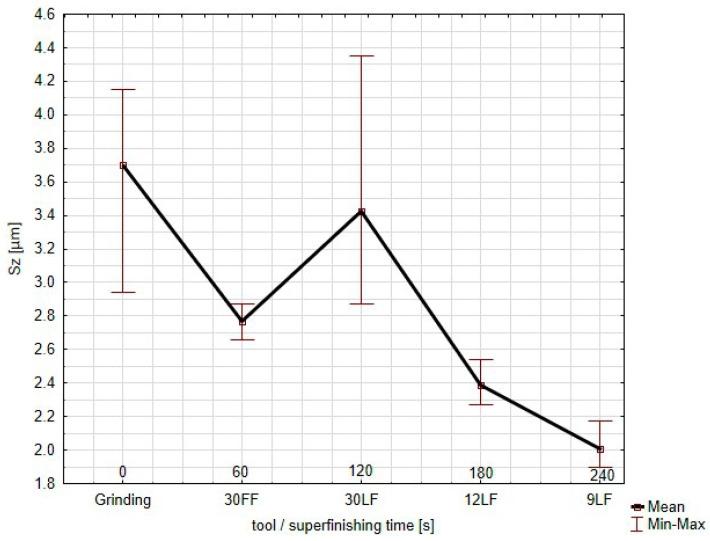
Values of the *Sz* parameter of the surface after successive machining stages.

The value of the Sa continues to decrease slightly with further refinement using the 30 LF film; the spread of values remains small. During the further superfinishing with the finer films (12 LF and 9 LF), the Sa parameter reaches a plateau at a very low value for the surface to attain a highly polished uniform finish. The minimal error bars further prove the excellent consistency of the surface roughness in the different locations during this stage. The Sa parameter shows that overall, each successive finishing tool used decreased the surface roughness uniformly. The minimum–maximum spread improved after each step, noticeably in the last stages of the process. The Sz parameter, representative of the maximum height of the surface, measured from the grinding to the use of increasingly finer superfinishing films (30 FF, 30 LF, 12 LF, and 9 LF) is shown in this graph (Figure 9).

Sz expresses the peak-to-valley distance of the highest peak and the deepest valley on the measured surface and characterizes the extremes of the overall surface roughness. The curve exhibits averages, while min–max bars represent the results dispersion for the three different measurement locations. As for the Sz, after the grinding, the value is relatively high; it presents great peaks and valleys on the surface. Large variations and surface irregularities are present, which in turn reflects in the wide min–max bars for such an initial rough process. When the application of the 30 FF film takes place, the Sz decreases abruptly, indicating a significant decrease in the very high features on the surface. The min–max bars become narrower with the improved homogeneity in the measured areas. Curiously enough, when the 30 LF film is applied, the Sz increases slightly and the min–max bars widen again; this may indicate that while the surface is being refined at this stage, it might also retain some deeper features or new ones during this intermediate stage. Such a phenomenon may not only result from the nature of the abrasive coating on the tool but also from the possibility of individual grains being torn out of the binder, which, as loose particles moving at high speeds, can uncontrollably generate single deep scratches. This effect is utilized in processes involving loose abrasive machining [52]. However, with the application of the finer films (12 LF and 9 LF), the Sz parameter decreases sharply and continuously, especially after the 12 LF, with the final measurement made by the 9 LF film showing the lowest maximum height. The min–max bars also shrink, showing the greater uniformity of and less variation in the surface roughness. The Sz parameter confirms that while the finishing process efficiently reduces the maximum surface roughness, during the 30 LF stage, there is a minor increase, presumably due to some specific interactions between the tool and material. In the final stages of the superfinishing, especially after using the 9 LF film, the surface becomes very refined with a low roughness and excellent uniformity for the different measuring points.

**Figure 10:**
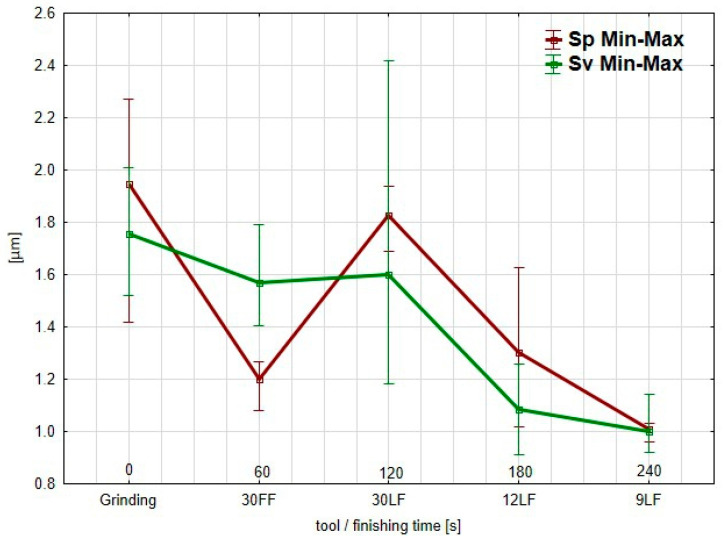
Relationship between the *Sp* and *Sv* parameters of surfaces smoothed with successive abrasive films.

The analysis of the Sp and Sv parameters (Figure 10), keeping in mind that Sz = Sp + Sv, allows noticing that the deterioration of the surface after the treatment with the 30 LF film can be related to the peaks that form on the surface, as was indicated by the increase in the Sp parameter. Evidence of this could be that the FF-type abrasive film is super effective in removing peaks from the surface. In such a type of film, large spaces between grains facilitate the effective removal of material. However, the more efficient LF-type films removed deep scratches from the surface, which was reflected by the reduction in the Sv parameter, while they were slower to eliminate the peaks. This may be because the spaces between grains are much smaller in these films. In the case of titanium treatment, this may be an important factor, as the material properties of titanium tend to generate clogging. Having a tool with increased spacing between grains would probably ensure better performance, as there would be less chance of clogging and improved material removal efficiency. From the graph, it is seen that both the parameters Sp and Sv are relatively high after the grinding, thus demonstrating sharp surface irregularities. The 30 FF film application decreases both the Sp and Sv, with the highest drop seen in the Sp, therefore improving the peak elimination by using this film. Further, for the 30 LF film, the value of the Sp increases, which was not expected, while the Sv remains quite stable, confirming the presence of peaks in this stage of the surface finishing.

Meanwhile, for the finer films, the Sp and Sv continuously decrease in a very regular way for the 12 LF and 9 LF; the 9 LF resulted in the most uniform and smooth surface because both the peaks and valleys were minimized. This suggests that FF films have a high efficiency in peak removal because of their grain distribution, and LF films, with a closer grain spacing, serve well in lowering valleys but might face difficulties in lowering peaks, especially on certain materials such as titanium, which tend to clog more frequently.

Standardized parameters of the description of surface geometry, though in common use, frequently give incomplete information about the real properties of such a surface. The majority of surfaces are designed to cooperate with other machine parts. In this case, standard parameters often fail to capture the features of possible contact areas, which may be important from a functional viewpoint. Therefore, new methods of surface description should be considered, which could be more useful in contact analyses. Complementing the classical approach, a detailed analysis of the surface elevations considered as the potential contact areas was performed. The cut-off surfaces used during this analysis gradually moved from the highest point of the analyzed surface downwards. In each “cut-off”, the elevations above the chosen plane were studied, which allowed the determination of the areas of possible contact between the interacting surfaces with more accuracy.

**Figure 11:**
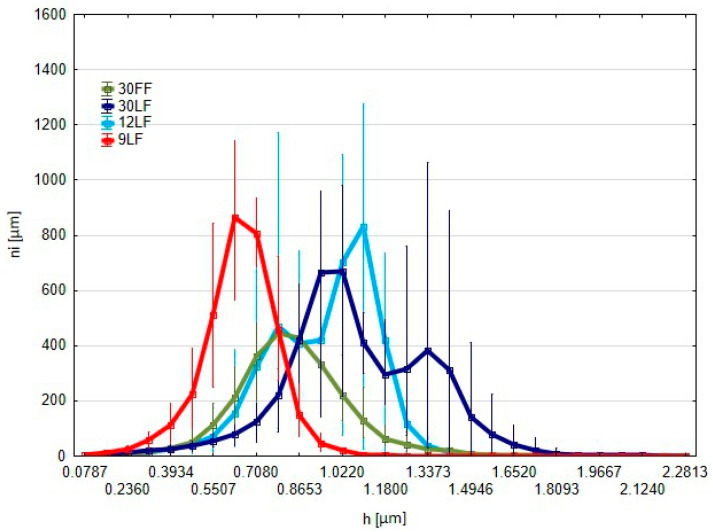
Graph of the number of islands *ni* raised above the cut-off plane *h* as a function of the cut-off plane position, where the value is measured from the highest point of the surface.

Based on the graph showing the distribution of the number of protrusions above the reference plane according to their height for the different abrasive films used for the surface finishing (Figure 11), several important features can be noticed.

The *Y*-axis shows the number of points where the surface rises above the reference plane—that is, the number of protrusions—which could be interpreted as contact points. The *X*-axis represents the distance in micrometers between the cut-off plane and the highest peak of the surface. The results show that, among the three films, the 9 LF is shifted furthest to the left. That would mean, in the surface smoothed with this film, the amount of protrusions with low height values is the highest. That would provide the most favorable tribological properties among the various films tested by providing more contact points and a more uniform protrusion. In the case of the 12 LF film, the distribution is much more scattered; here, too, the number of protrusions is higher in the lower height ranges, which can further contribute to an improvement in surface properties. Conversely, for the 30 LF film, this distribution is extended and the protrusions peak at higher height values; this means fewer contact points with larger protrusions result in poorer tribological properties compared with those of the 9 LF and 12 LF films. Because the 30 FF film has the broadest distribution of protrusions and the highest height values, this film produces a surface with the fewest contact points and the largest protrusions. Such a topography is not desirable from a tribological point of view. The best tribological properties were realized for the 9 LF film due to it providing the maximum number of contact points with the smallest protrusions. Similarly, coarser films, such as the 30 FF or 30 LF, provide larger protrusions and fewer contact points, which is less favorable from a tribological point of view. In other words, it means that films with finer grains, especially the 9 LF one, are suitable in those applications where high wear resistance and a much more homogenous contact surface are required.

**Figure 12:**
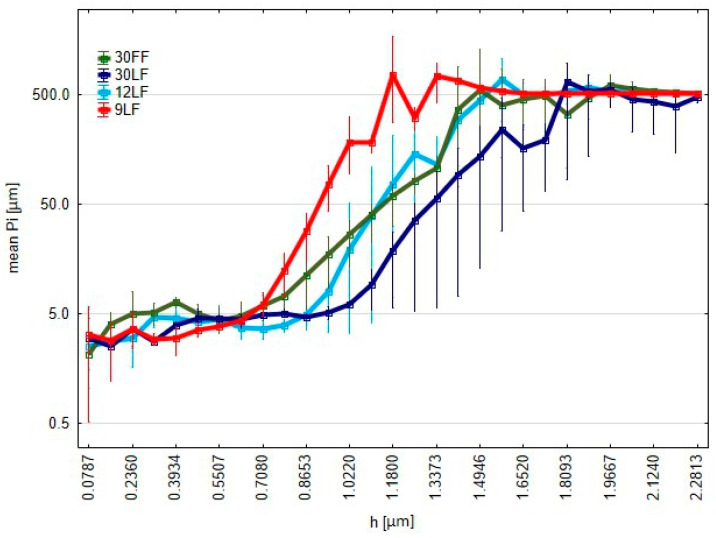
Graph of the average perimeters of islands *Pi*, i.e., cross–sections with the cut-off plane, as a function of the cut-off plane position *h.*

These plots depict the perimeters of the islands that rise above the cut-off plane, which is at a distance h from the highest point on the surface (Figure 12). It can be further observed from the analysis that the perimeters of the 9 LF film increase the most quickly, which is a desirable phenomenon, because this means that the surface treated with this film contains many contact points in large numbers, indicating more uniform and stable protrusions rather than single peaks. In contrast, the poorest performance is seen from the 30 LF film, where perimeter increases more slowly, perhaps indicating a poorer surface topography. This kind of analysis enables the precise assessment of how the different films affect the properties of the surface after treatment.

**Figure 13:**
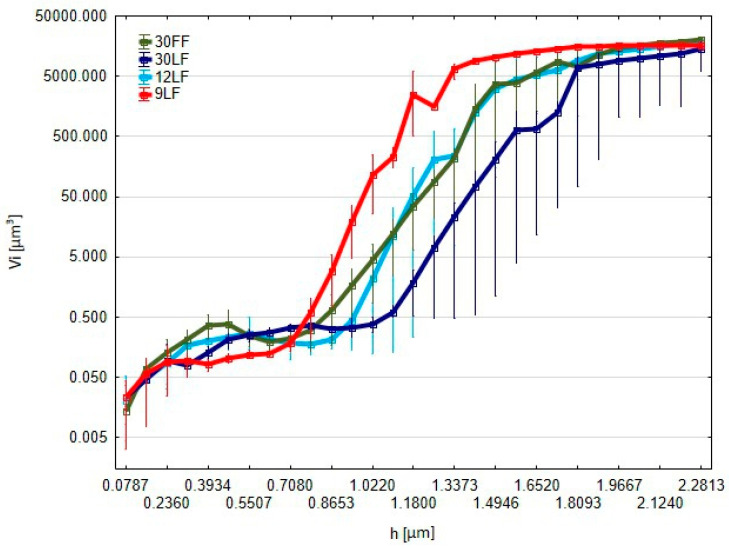
Graph of the average volumes of islands *Vi* above the cut-off plane positioned at a distance *h* from the highest point of the surface.

The red curve in this graph corresponds to the 9 LF film and represents the mean volume of the elevations *Vi* (Figure 13), reaching values of around 5000 cubic micrometres with higher parameter values *h*. In the 9 LF film, the increase in volume is much more homogeneous compared to the other films, probably implying its superiority in view of the process stability during the material removal. Such a consistent increase would most likely mean that the 9 LF film may be preferred when the treatment of the surface should be made precisely and uniformly in some processes. The 9 LF film exhibits the largest and most uniform volume increase, resulting in surfaces with enhanced precision in some superfinishing processes, while the 30 LF, 30 FF, and 12 LF films offer clear advantages in conditions requiring process stability or reduced material removal, imparting specific characteristics to the surfaces.

**Figure 14:**
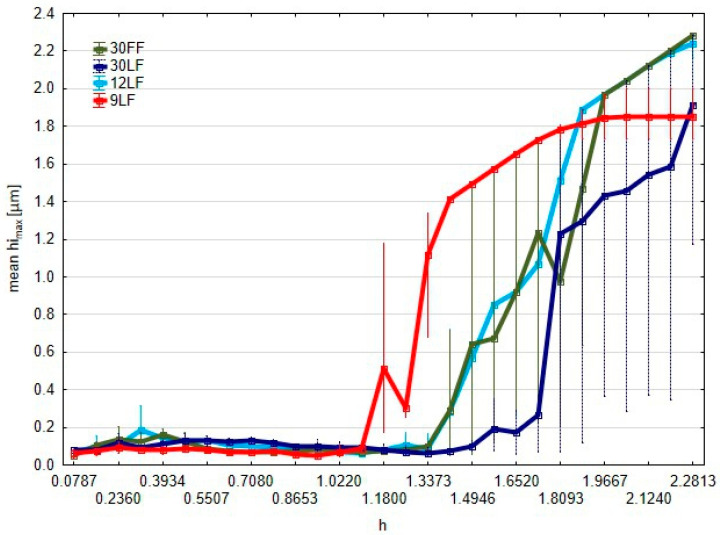
Graph of the average maximum heights of islands (their peaks) *hi_max_* as a function of the distance *h* of the cut-off plane position.

From the results shown in Figure 14, it can be remarked that the maximum heights of the elevations *hi_max_* above the cutting plane change greatly with regard to the type of superfinishing film used. Indeed, the 9 LF gives rise to the highest elevations, rapidly reaching significant values as the *h* parameter increases. A threshold value above these values increases very sharply in this graph. Here, the graph can be understood to imply the capability of the film for aggressive material removal and hence to give significant height elevations on the surface. These correspond, when compared to the volume of the elevations, with an increase in the volume uniformity of the 9 LF with the maximum heights of the elevations. The uniformity is indicative of the process stability, and the 9 LF film develops not only a substantial quantity of material on the surface but also elevations of substantial height, which might be relevant in the context of precision surface treatment, where the uniformity of the surface finish is imperative. These findings regarding the maximum height of elevations above the cutting plane all align with the previous analyses of the volumes and perimeter of the elevations. Of those, the 9 LF is an outlier in both the extent of the elevations removed and in the consistency of the process.

This is due to the fact that standardized parameters may not always depict the level of surface smoothing; analysis of the elevations above the cutting plane can describe the potential contact areas of a surface topography created for operational applications. A surface smoothness evaluation coefficient (*c_e_*) has been developed, which takes into account the level of surface finishing based on the number of surface contacts and the maximum number at any given level. The value of the cutting plane position is preferably low, as a low position improves the operational characteristics of such a surface. Additionally, the number of elevations should be greater because such a structure is capable of positively influencing the retention of lubricants during future operation. For instance, the intentional crossing of machining tracks is utilized in the honing process of cylinders because this configuration has been shown to enhance the retention of lubricating films on these surfaces.

Further, the results of the calculational estimations for the proposed surface smoothing efficiency coefficient are summed up in Table 2. The maximum value, 50.44, corresponds to the 9 LF film; it is possible to state that the given film has smoothed the surface most effectively. The minimum value of the smoothing capability corresponds to the 30 FF film; that is completely explained, since this film was mainly used during the preparatory treatment of the surface.

In particular, the index of surface smoothness developed herein is very significant since it will allow detailing the effect of the surface characteristics on performance in field applications. The proposed index targets the points of contact and their distribution, considering both aesthetically smooth surfaces and those performing well under loads. Indeed, this relationship between geometry and operational performance can be critical when the application relies on lubrication. The data for the 9 LF film, moreover, suggests that the finely textured nature of the surface created increases lubrication retention to reduce wear and increase the service life of components. This is beneficial in an industrial setting when heavy machinery is often involved and requires lubrication to avoid overheating and possible damage. Therefore, the choice of finishing film must correspond to those indices in order to optimize the surface properties and ensure the long service life of the mechanical components. In short, an index estimating the surface smoothness from contact points and their maximum occurrence introduces a good insight into the possible performance of the treated surfaces. The results clearly underline the superiority of the 9 LF film for achieving superior smoothing, while the role of the 30 FF film is restricted to the initial treatment only, drawing on the selection of the right finishing method for appropriate applications.

It is evident that the proposed surface smoothness coefficient does give a more complete rating than the traditional roughness parameters, such as Ra, considering only amplitude-type measurements. Traditional measurements can be given for the overall amount of variation in surface height, but they do not take into account the functional aspects of surface topography with respect to contact points, which are usually very relevant for practical applications in which wear resistance and the retention of lubricants are very important. The new coefficient gives great prominence to the amount and distribution of these contact points, hence giving a more readable understanding of surface topography performance in functional situations. Used in addition to traditional metrics, this approach allows deeper analysis, ensuring the surface is not only looking smooth but also working well under extreme conditions. This coefficient is an enhancement in this frame, which gives a more accurate process optimization and allows for better decision-making on tool and parameter selection for finishing the surface, because the imposed load on the components is very high, or requires effective lubrication, especially in aerospace and industrial machinery.

### 3.3. SEM Analysis of Machining Products in the Smoothing Process Using Abrasive Films

**Figure 15:**
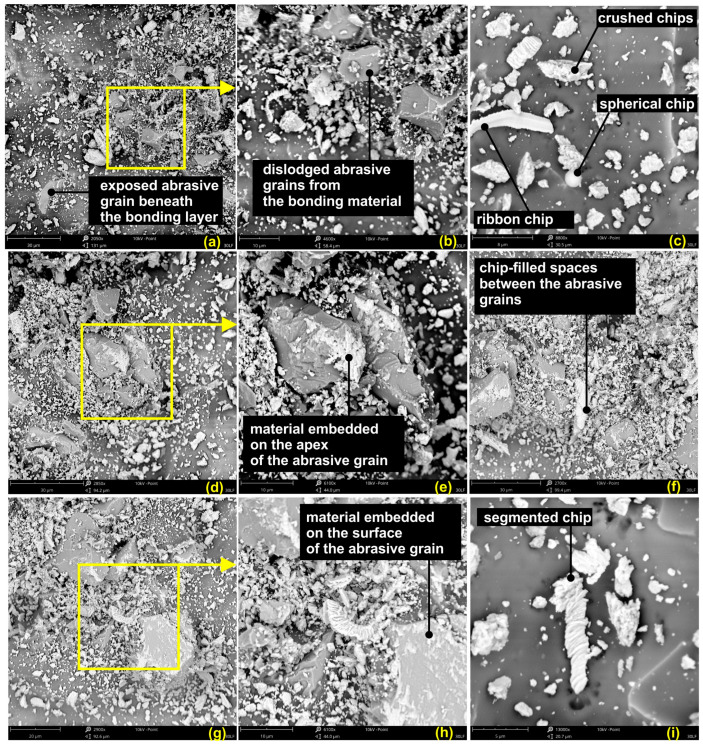
SEM images of the 30 LF (**a**–**i**) abrasive film surface after the superfinishing process.

In order to further understand the superfinishing process of the surfaces with a titanium alloy, an analysis of tool surface post-processing was performed. In Figure 15, the results are presented for the surface analysis for the 30 LF tool. Comparing the images of the tool after processing with the images of new tools, an observation can be made: during the initial finishing phase, the binder was already worn from the working surfaces of the abrasive grains in the machining zone. In Figure 15a,d, respectively, exposed abrasive agglomerates can be observed in the magnifications in Figure 15b,e, correspondingly. It is certain that these grains were cutting because the material from the workpiece was still attached to the tips of these grains. Moreover, it should also be pointed out that the investigated titanium alloy has a tendency to adhere to the tool surface (Figure 15g,h) or to the cutting edge of the grain. Additionally, the interspaces between the grains are also filled with removed workpiece material which undergoes plastic deformation and forms an agglomerate (Figure 15f).

The nature of the chips produced ribbon-like chips with segmented structures, observed Figure 15i, along with numerous fragments of broken chips. This feature is typical for titanium in the course of the smoothing process, while other materials, for example, superalloys, show more proneness for ribbon-like chips to appear during microfinishing with abrasive films. The fragmented form of the chips, hence, is not representative for the characteristic of the machining process but rather for the material being treated. Further, a few spherical chips were observed, as shown in Figure 15c, which is evidence of high temperatures in the machining zone. These high temperatures cause the melting of the material, which afterwards resolidifies into the form of spherical chips. The presence of such features underlines the complexity of the superfinishing process for titanium alloys; temperature, material properties, and tool wear are the factors that determine the outcome of the machining process through mutual interactions.

**Figure 16:**
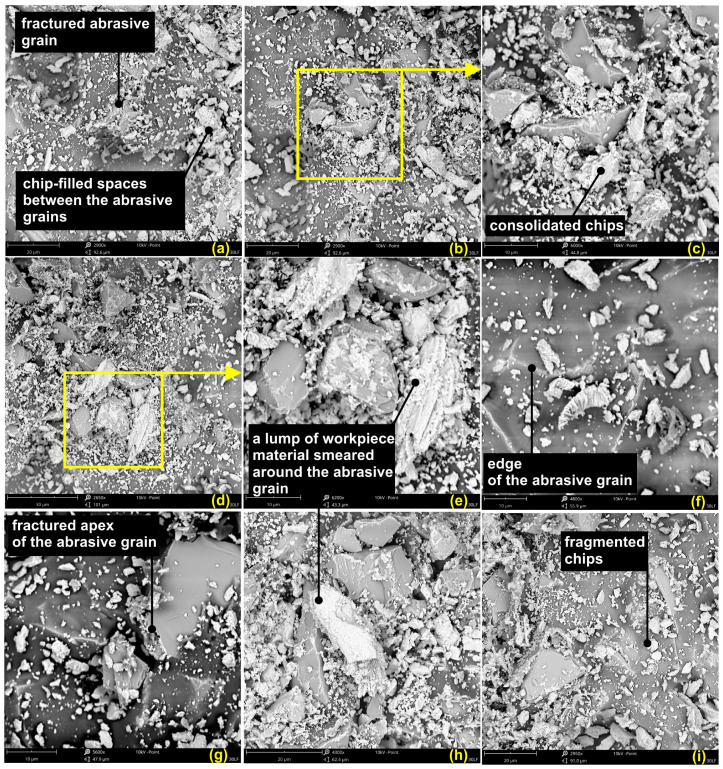
Scanning electron microscope (SEM) images of the 30 LF (**a**–**i**) abrasive film surface following the superfinishing procedure.

Further characterization by the means of SEM microscopy of the surfaces of the abrasive films are given in Figure 16.

It is to be noticed that a relatively large number of the abrasive grains did not take part in the machining process. In the image in Figure 16f, only the tips or edges of the abrasive grains appear above the binder, while the rest of their parts remain covered with the binder layer, showing those grains did not take part in the process. What is more, the abrasive grains can be destroyed during the machining process, as shown in Figure 16a: an abrasive grain has fractured into half. Figure 16g shows a chipped tip of an abrasive grain. All these phenomena are undesirable because the detached abrasive grain or its pieces will scratch the workpiece surface deeply.

Figure 16b–e further confirm that the material removed from the workpiece also tends to adhere to the surface of the film and become embedded around the abrasive grains. Adhering debris will badly affect the performance of the abrasive film by allowing friction and altering the intended cutting action of the grains, thus seriously reducing the tool’s ability to smooth surfaces effectively. It should be realized that in superfinishing, the effectiveness of the process depends not only on active participation of the abrasive grains but also on structural integrity and the removal of debris. In other words, the accumulation of this processed material around the abrasive grains may obstruct the cutting edges and result in less effective machining, compromising the quality of the finished surface. In this respect, one should consider that the increased friction, caused by the built-up swarf, deteriorates the quality of the treated surface, while it contributes to the premature wear of the tool. If there is not enough swarf removal, the overheating of the abrasive layer takes place. The latter reduces the durability of the abrasive layer and the overall efficiency. Uncontrolled and long-lasting residual material accumulation leads to the micro-injuries of the tool. It can gradually worsen its working potential. It would, therefore, be of essence that this concern be effectively taken care of through the selection of appropriate process parameters–pressure and machining speed–which will allow the abrasive particles to remain functional and effective in their action throughout the operation. The requirement of optimizing the superfinishing process, therefore, demands an understanding of the interaction between the abrasive particles, the workpiece material, and the resulting debris. Adopting the right methodology helps in not only restraining the build-up of the material being processed but also gives direct beneficial consequences to the improved surface finish quality and tool longevity, particularly under aggressive industrial use. Therefore, a proper understanding of the interactions among the abrasive grains, binder, and workpiece would be a necessary ingredient for the optimization of the superfinishing process. Consequently, this might lead to considerations over possible solutions to address the problems about grain inactivity and material adherence. Post-processing analysis of the 30 LF tool surface provides the necessary information related to superfinishing of titanium alloys. The wear patterns, chip morphology, and evidence of adhesion suggest that the control of process parameters is important to optimize surface quality and performance in practical applications. Such an understanding will go a long way towards enhancing the efficiency and effectiveness of the superfinishing process for titanium alloys and, therefore, their performance under challenging industrial conditions.

## 4. Summary and Conclusions

The current study focuses on surface finishing efficiency regarding titanium alloy Grade 5, Ti–6Al–4V, after superfinishing with abrasive films having different grain sizes. It determines the superfinishing process to obtain a smooth, high-quality surface with a wide range of applications in demanding industries like aerospace and biomedical fields. In the experiment, four different types of abrasive film were considered: 30 FF, 30 LF, 12 LF, and 9 LF. The aim was to establish their efficiency in reducing surface roughness and enhancing overall finish quality. Various advanced measurement techniques, including SEM microscopy and surface topography analysis, were performed in minute detail to understand the abrasive grain and binder interactions with the titanium alloy. It was shown that the films with a smaller grain size, among which the 9 LF had the smallest, provided not only the lowest surface roughness but also allowed a more homogeneous distribution of contact points, hence improving the tribological properties of the surfaces. The larger nominal grain size films like the 30 FF and 30 LF are much less effective films because of their larger protrusions and fewer contact points. In this investigation, a coefficient for the estimation of surface smoothness was newly proposed, which considers the distribution of contact points as well as the number of contact points. The proposed index can represent the core of innovation, providing interesting hints on the efficiency of the superfinishing process, and giving further contributions to the refinement of machining technologies in titanium alloys.

- The 9 LF abrasive film demonstrated the highest efficiency regarding surface smoothing, with a coefficient of 50.44, compared to the other films. Therefore, it is the most effective for a top-quality surface finish.
- The abrasive films with finer grains gave very smooth surfaces, with the 9 LF film producing almost flawless finishes. In contrast, the coarser grained films gave rise to more marked surface protrusions that seriously deteriorated the surface quality.
- The 9 LF film provided the best tribological properties due to its ability to maximize the contact points while minimizing protrusions. It is highly suitable for very demanding applications concerning high wear resistance and superior durability.
- There was debris that clogged the abrasive films during the machining process; this significantly reduced their performance by increasing friction, hence lowering their effectiveness in smoothing. Good debris management is needed for optimum film performance.
- High temperature in the machining zone melted the material and, with it, formed spherical chips. Such chips may be problematic for the superfinishing process or enlarge the effects on the surface consistency, which requires essential temperature control.

## Figures and Tables

**Table 1:** Processing parameters for the experiments.

Workpiece Material	Pressure Roll Hardness	Pressure Force	Tool Speed	Workpiece Speed	Oscillation Frequency	Processing Time
Titanium Alloy Grade 5 (Ti–6Al–4V)	50°Sh	50 N	160 mm/min	10 m/min	80 Hz	360 s

**Table 2:** Calculation for the proposed surface smoothing efficiency coefficient.

Tool	$n_{i_{max}}$	${\left(\frac{hmax}{Sz}\right)}_{nimax}$	c_e_
30 FF	458.66	0.31	38.38
30 LF	671.33	0.36	43.20
12 LF	833.31	0.48	41.65
9 LF	863.00	0.34	50.44

## Data Availability

The original contributions presented in the study are included in the article, further inquiries can be directed to the corresponding authors.
